# The Evaluation of Olfactory Function in Patients With Schizophrenia

**DOI:** 10.5539/gjhs.v7n6p319

**Published:** 2015-04-23

**Authors:** Soleimani Robabeh, Jalali Mir Mohammad, Ahmadi Reza, Badri Mahan

**Affiliations:** 1 Shafa Hospital, Guilan University of Medical Sciences, Iran; 2 Sinonasal Disease Research Center, Amiralmomenin Hospital, Guilan University of Medical Sciences, Iran; 3 Medical Faculty, Guilan University of Medical Sciences, Iran

**Keywords:** olfactory perception, pleasantness, schizophrenia, smell

## Abstract

The aim of this study was to compare olfactory threshold, smell identification, intensity and pleasantness ratings between patients with schizophrenia and healthy controls, and (2) to evaluate correlations between ratings of olfactory probes and illness characteristics. Thirty one patients with schizophrenia and 31 control subjects were assessed with the olfactory n-butanol threshold test, the Iran smell identification test (Ir-SIT), and the suprathreshold amyl acetate odor intensity and odor pleasantness rating test. All olfactory tasks were performed unirhinally.

Patients with schizophrenia showed disrupted olfaction in all four measures. Longer duration of schizophrenia was associated with a larger impairment of olfactory threshold or microsmic range on the Ir-SIT (P = 0.04, P = 0.05, respectively). In patients with schizophrenia, female subjects’ ratings of pleasantness followed the same trend as control subjects, whereas male patients’ ratings showed an opposite trend. Patients exhibiting high positive score on the positive and negative syndrome scale (PANSS) performed better on the olfactory threshold test (r = 0.37, P = 0.04). The higher odor pleasantness ratings of patients were associated with presence of positive symptoms.

The results suggest that both male and female patients with schizophrenia had difficulties on the olfactory threshold and smell identification tests, but appraisal of odor pleasantness was more disrupted in male patients.

## 1. Introduction

Patients with schizophrenia have selective impairments in the temporolimbic and frontal lobe regions of the brain. Olfaction is closely associated with these neuroanatomical regions, and is intimately related to the affective and mnemonic functions that they subserve (Turetsky et al., 1995). The significance of olfactory deficits relates to the neuroanatomical overlap between brain areas associated with the olfactory processing and the neuropathology of this disorder. Different subgroups of schizophrenia patients might express characteristic changes in the rating range of olfactory performance estimates depending on their symptom profiles.

Deficits in smell identification, considered to be connected to central olfactory mechanisms, are widely reported in schizophrenia (Atanasova et al., 2008). It was shown this impairment to be correlated with deficits in motivated behavior and emotional expression as well as with impaired verbal and nonverbal memory (Compton et al., 2006; Malaspina et al., 2002; Seckinger et al., 2004). Olfactory identification deficits may be associated with negative symptoms (Brewer et al., 1996; Malaspina et al., 2002; Malaspina & Coleman, 2003; Corcoran et al., 2005). Although informative, smell identification assessment may not provide a full picture of the olfaction abnormalities in schizophrenia. In fact, a number of studies have shown that smell identification deficits can exist in people with intact olfactory sensitivity (Kopala, Clark, & Hurwitz, 1989; Kopala, Clark & Hurwitz, 1993; Striebel, Beyerstein, Remick, Kopala & Honer, 1999).

Other aspects of olfaction studied in schizophrenia include threshold, discrimination, familiarity/recognition, intensity and pleasantness; however, the findings have been variable (Atanasova et al., 2008). One interpretation of this could be that the deficits in smell identification in schizophrenia reflect abnormalities in central processing, whereas the supposedly unchanged threshold reflects normal peripheral processing. Keller and Vosshall (2004) demonstrated that odor threshold is strongly influenced by higher level processing, even though it can be considered to be a peripheral system.

Crespo-Facorro et al. (2001) found that patients with schizophrenia had diminished capacity to experience pleasure within the olfactory domain. Hudry et al. (Hudry, Saoud, D’Amato, Dalery, & Royet, 2002) showed that judgment of odor intensity did not differ between patients with schizophrenia and control subjects. Similarly, Moberg et al. (2003) found that male patients with schizophrenia differed from control subjects in their assignment of odor pleasantness to amyl acetate, despite being nearly identical to controls with regard to intensity judgment. Doop and Park (2006) found that abnormal pleasantness judgments were positively correlated with the affective flattening subscale of the Scale for Assessment of Negative Symptoms (SANS). In a study by Plailly et al. (Plailly, D’Amato, Saoud, & Royet, 2006), patients with schizophrenia had diminished familiarity with odors and rated odors deemed pleasant by healthy subjects less favorably. Conversely, others have reported increased odor pleasantness ratings in patients with schizophrenia (Cumming, Matthews, & Park, 2011; Rupp et al., 2005) or pleasantness ratings restricted to the high end of the pleasantness spectrum in contrast to broader ratings observed in controls (Doop & Park, 2006).

Studies in healthy people that have used intensity and pleasantness ratings of some olfactory stimuli (e.g., amyl acetate, furfural), showed that pleasantness ratings are highest (i.e., most pleasant) at weak concentrations and decline progressively (i.e., become more unpleasant) as odorant concentration increases (Henion, 1971; Moskowitz, & Gerbers, 1974). Across these studies, disparate odors have been used to probe odor processing, and the different chemical components of these odors and variations in the circuits they activate may have produced confounding results (Doty, McKeown, Lee & Shaman, 1995). In an attempt to minimize these confounds, several researchers have used the amyl acetate test, administering the odorant birhinally at four suprathreshold concentrations (Kamath, Moberg, Kohler, Gur & Turetsky, 2013; Moberg et al., 2003). Tests such as this allow researchers to probe bi-dimensionals ratings of pleasantness using a single odor (in other words, pleasantness-unpleasantness and intensity), thus avoiding the potential confounds posed by using several different odors.

Despite the relationship between olfactory and schizophrenia, there has been little direct investigation of the different aspects of olfaction in Iranian patients. A large body of research in olfaction has shown that cultural variation may lead to the modulation and development of odor preferences. Chrea et al. (2004) evaluated the effect of culture on the relationship between psychological dimensions underlying odor perception and odor categorization in normal participants. They concluded that Western people and Asian people think differently and differed in their judgments for several perceptual dimensions. Ayabe-Kanamura et al. (1998) observed clear differences between Japanese and German women in pleasantness ratings. The researchers postulated that cross-cultural difference in tolerance to or acceptability of particular odor components may be existed. Also studies that evaluate different components of olfactory processing and symptomatology may be crucial to improve the understanding of the causes for social function deficits in these individuals, and guide the development of new person-targeted treatment. Therefore, we conducted a comprehensive assessment of olfactory function in patients with schizophrenia and a group of healthy subjects.

## 2. Materials and Methods

### 2.1 Participants

Participants were recruited by the Guilan University of Medical Sciences and placed into 1 of 2 groups as follows: (1) patients with schizophrenia (n = 31) who met the Diagnostic and Statistical Manual of Mental Disorders, Fourth Edition, Text Revision (DSM-IV-TR) (American Psychiatric Association, 2000) diagnosis of schizophrenia, and (2) healthy subjects (n = 31).

All participants underwent a psychiatric interview (Structured Clinical Interview for DSM-IV, Patient or Nonpatient Edition) (First, Gibbon, Spitzer & Williams, 1997a), and a detailed medical history was taken, including a physical examination and laboratory testing by a psychiatrist (S.R.). A resident in psychiatry (A.R.) rated patients on the Brief Psychiatric Rating Scale (BPRS) (Overall & Gorham, 1962), Positive and Negative Syndrome Scale (PANSS) (Kay, Fiszbein, & Opler, 1987), and the Hamilton Psychiatric Rating Scale for Depression (HAM-D) (Hamilton, 1960). Control subjects were assessed for a DSM-IV Axis II disorder with the Structured Clinical Interview for DSM-IV Personality Disorders (First, Gibbon, Spitzer, Williams & Benjamin, 1997b), and were free of any axis I diagnosis, axis II cluster A personality disorder, and family history of psychiatric illness.

Individuals were excluded from participation if they had a history of neurological disorder, loss of consciousness, head trauma, mental retardation, or substance dependence, or if they were abusing a substance (determined by a urine drug screen), or had the presence of a medical condition that could alter cerebral or olfactory functioning at the time of the study. All patients were stable outpatients at the time of testing, and were on a regimen of antipsychotic medications (1 patient on first-generation antipsychotics (FGAs), 14 patients on second-generation antipsychotics (SGAs), and 16 patients on both). The daily dose of antipsychotic medication prescribed to each patient was converted into milligram equivalents of chlorpromazine according to conversion factors derived from the literature (Bazire, 2005; Woods, 2003; Kane et al., 1998). The Chlorpromazine dose equivalents (CPZeq) is a measure of the relative antipsychotic potencies of neuroleptics. They are generally expressed as a ratio, relative to the arbitrary value of 1, which corresponds to the antipsychotic effects of chlorpromazine. Total CPZeq was constructed by calculating a total daily dose of each antipsychotic listed in the medical file. Then each converted antipsychotic-specific CPZeq amount is added to arrive at a total dose. Classification of antipsychotics in FGAs or SGAs and the computation of CPZeq were done according to the literature and listed in Table 1.

**Table 1:** Antipsychotic dosing equivalents

Drug*	Potency ratio	Antipsychotic equivalent doses
FGA		
Low-potency		
Chlorpromazine tablet	1.0	300.0
Thioridazine tablet	1.0	300.0^1^
Medium potency		
Perphenazine tablet	12.5	24.0^1^
High-potency		
Flupenthixol decanoate	70.0	4.2^1,3^
Haloperidol tablet	33.0	9.0^1^
Haloperidol decanoate	50.0	6.0^1,3^
SGA		
Aripiprazole tablet	13.3	22.5^2^
Clozapine tablet	1.0	300.0^1^
Olanzapine tablet	20.0	15.0^2^
Quetiapine tablet	1.3	225.0^2^
Risperidone tablet	66.0	4.5^1^

FGA: First-generation antipsychotics; SGA: Second-generation antipsychotics.

1 Bazire, S. (2005). *Psychotropic Drug Directory*. Fivepin Limited, Salisbury.

2 Woods, S. W. (2003). Chlorpromazine equivalent doses for the newer atypical antipsychotics. J. *Clin Psychiatry*, 64, 663-667.

3 Kane, J.M., Aguglia, E., Altamura, A. C., Ayuso Gutierrez, J. L., Brunello, N., Fleischhacker, W. W., et al. (1998). Guidelines for depot antipsychotic treatment in schizophrenia. European Neuropsychopharmacology Consensus Conference in Siena, Italy. *Eur Neuropsychopharmacol, 8*(1), 55-66.

Goel and Grasso (2004) observed time-of-day differences in response to various odors. Albrecht et al. (2009) found that both odour threshold as well as pleasantness play an important role in the control of food intake and satiety has the effects on detection thresholds of food-related odor isoamyl acetate. Given the potential confounding factors, we conducted all tests in the morning (08:00–10:00 h) within at least 60 min after the meal. All olfactory tasks were performed unirhinally because in addition to exposing potentially important laterality differences, unirhinal testing eliminates the effects of birhinal facilitation, whereby olfactory performance is improved through secondary integration of the separate left and right nostril afferent inputs (Turetsky, Hahn, Borgmann-Winter, & Moberg, 2009).

Written informed consent was obtained from all participants. All study procedures were approved by the Institutional Review Board of the Guilan University of Medical Sciences. The study was conducted according to the Declaration of Helsinki on Biomedical Research Involving Human Subjects.

### 2.2 Olfactory Threshold Test

The olfactory threshold test battery consisted of 48 sniff bottles (Doty et al., 1995). Sixteen bottles contained n-butanol at different concentrations, with a maximum concentration of 4%. Serial dilutions (1:2 ratio) of n-butanol were prepared using water as a solvent. The remaining 32 bottles were blanks. Using a triple-forced-choice paradigm, detection thresholds were determined by employing a single staircase method as described by Hummel et al. (Hummel, Sekinger, Wolf, Pauli & Kobal, 1997). The detection threshold was defined as the mean of the last four out of seven staircase reversal points. The scores ranged from 0 to 16. Bottle 1 (4% n-butanol) and bottle 16 had the strongest and the weakest dilution of n-butanol, respectively. In this scoring system, a higher score means a lower threshold (good performance).

### 2.3 Smell Identification Test

To test the function of each subject’s olfactory system, we used the Iran Smell Identification Test (Ir-SIT, Saba Tajhiz Sabalan LLC), which is commercially available. This battery kit includes 24 forced choice tests, similar to the University of Pennsylvania Smell Identification Test (UPSIT). The test was standardized in the Iranian population and shows high test-retest reliability (r = 0.93). The Ir- SIT manufacturer rates scores below 10 indicative of anosmia, scores of 10-19 indicative of microsmia, and scores of 20-24 indicative of normosmia.

### 2.4 Odor Intensity and Odor Pleasantness Rating Tests

Measures of odor intensity and pleasantness were assessed using the Suprathreshold amyl acetate Odor intensity and Odor pleasantness rating tests (Moberg et al., 2003). Subjects were presented with 100-ml glass sniff bottles with 4 concentrations (−1.00, −2.00, −3.00, and −4.00 log vol/vol, from strong to weak intensity) of amyl acetate, with light mineral oil as the diluent. The subjects rated the perceived intensity and pleasantness of the odor on separate 5-point Self-Assessment Manikin scales (Lang, 1985). The 4 odor concentrations were presented 5 times in a counter-balanced order to each nostril, for a total of 20 trials in each rating condition. Previous research has suggested a test-retest reliability of 0.75 or greater for similar tasks (Doty, 1995).

### 2.5 Data Analysis

We assessed outliers in variables. When this dilemma existed, we run the analysis with and without the outliers. If difference was little, we ignored the presence of the outliers. Otherwise, we transformed variables as logarithms and described conclusions based on the results for the transformed variables. If transformed variables didn’t have normal distribution, we run analyses with using non-parametric tests. Group differences in age, education, smoking, and parental education were assessed using a t - test. Pearson chi-squared tests were used to determine group differences for sex and handedness. Repeated measures multivariate analyses of covariance (MANCOVAs) were conducted for intensity and pleasantness ratings. Left and right nostril ratings were repeated measures factors, with group and sex as between-subject factors. Smoking (packs/day) was included as a covariate in all analyses. Significant MANCOVA effects were followed by univariate analyses examining pairwise group contrast on the individual measures. Within the schizophrenia group, relationships between pleasantness ratings and clinical attributes (duration of illness, age of onset, negative and positive symptoms, and chlorpromazine equivalents) were measured using Pearson’s correlations (r). Statistical analyses were performed using Stata 12.0 (StataCorp, College Station, Texas).

## 3. Results

### 3.1 Participant Characteristics

Participant demographic and clinical data are presented in Table 2. There were no differences between the patients with schizophrenia and healthy controls in the percentage of male and female participants, mean age, or handedness. As expected, patients had lower educational levels than the healthy subjects did (P < 0.001). We also observed lower parental educational levels in the schizophrenia group. The percentage of patients with schizophrenia and control subjects that were smokers was 58.06% and 54.84%, respectively. There was no significant difference in smoking burden (defined as pack years) between the two groups (P = 0.29). As seen in Table 3, the results of various olfactory tests did not differ each other when odor was presented to the left or right nostril.

**Table 2:** Demographic and clinical characteristics of schizophrenic patients and healthy subjects

Characteristics	Schizophrenic Patients (*n* = 31)		Control subjects (*n* = 31)		*P* value*
	
	*N*	%	*N*	%	
Sex					0.57
Males	24	77.4	21	67.7	
Females	7	22.6	10	32.3	
Handedness					0.21
Right	27	87.1	22	71	
Left	4	12.9	9	29	
	Mean	SD	Mean	SD	
Age (years)	37.61	11.14	38.94	13.17	0.69
Education (years)	9.77	3.59	13.94	4.30	0.001
Mother’s education (years)	6.84	6.53	10.10	6.11	0.05
Father’s education (years)	3.06	4.92	7.03	6.49	0.009
Pack-day	0.52	0.95	0.26	0.35	0.17
Age of onset (years)	25.97	9.04				
Illness duration (years)	11.68	8.41				
BPRS total score	56.29	11.39				
Negative scale of PANSS	27.32	8.93				
Positive scale of PANSS	28.90	4.09				
HAM-D total score	18.39	6.05				
Chlorpromazine equivalents	416.94	135.57				

*Note.* BPRS, Brief Psychiatric Rating Scale; HAM-D, Hamilton Psychiatric Rating Scale for Depression; PANSS, Positive and Negative Syndrome Scale.

* All analyses (except for sex and handedness) were performed with *t* test. For sex and handedness, two groups were compared with Chi square test.

**Table 3:** The results of various olfactory tests in schizophrenic patients and healthy subjects

Characteristics	Schizophrenic Patients (*n* = 31)			Control subjects (*n* = 31)			*P* value*
	
	Right nostril	Left nostril	Mean±SD	Right nostril	Left nostril	Mean±SD	
Olfactory Threshold test	9.02	8.70	8.86±2.42	13.26	13.17	13.21±1.66	0.001
Smell Identification test	15.88	16.39	16.13±4.62	19.88	21.16	20.52±3.14	0.001
Pleasantness rating to the strongest odor	13.67	13.43	13.55±8.65	6.77	6.29	6.53±1.40	0.001
Pleasantness rating to the weakest odor	10.84	10.95	10.89±5.76	14.82	14.37	14.60±2.15	0.001
Intensity rating to the strongest odor	23.08	23.05	23.06±2.79	24.20	24.17	24.19±1.75	0.06
Intensity rating to the weakest odor	7.92	8.21	8.06±3.01	6.15	6.11	6.13±2.02	0.004

* All analyses in two groups were performed with *t* test.

### 3.2 Olfactory Threshold Test

There was a significant difference between the mean olfactory threshold of patients with schizophrenia and healthy subjects (P < 0.001). Longer duration of disease was associated with a larger impairment in olfactory threshold (r = −0.37, P = 0.04). However, patients that exhibited more positive symptoms (high positive score on the PANSS) scored better on the olfactory threshold test (r = 0.37, P = 0.04). There was no significant correlation between a patient’s olfactory threshold and their age, age of onset of disease, chlorpromazine equivalent dosage, or BPRS score.

### 3.3 Smell Identification Test

Using the Ir-SIT, we found a significant difference in mean test score between two groups (P < 0.001). Ir-SIT score in the schizophrenia group was significantly correlated with BPRS scores (r = −0.38, P = 0.03), but not with duration of illness or any other variables. Seventy-four percent of patients with schizophrenia scored in the microsmic range, compared with only 29% of healthy subjects (P = 0.001). In patients with schizophrenia, those that scored in the microsmic range had a significantly longer duration of illness than those that scored in the normosmic range (16.63 year versus 9.96 year; P = 0.05).

### 3.4 Intensity Ratings

**Figure 1:**
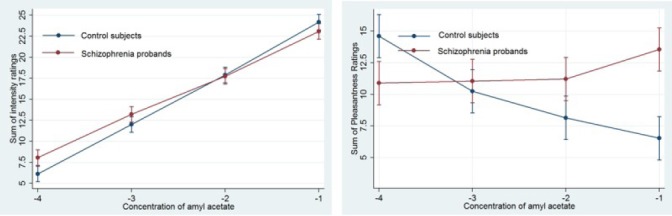
Odor intensity and pleasantness ratings (± 95% CI) for various concentrations (-4.00 to -1.00 log vol/vol) of amyl acetate in schizophrenic patients, and controls

**Figure 2:**
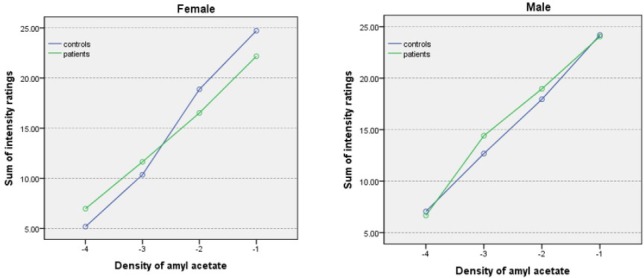
Odor intensity ratings by gender for various concentrations (-4.00 to -1.00 log vol/vol) of amyl acetate in schizophrenic patients and controls

Analysis of intensity ratings on the amyl acetate test revealed a significant main effect of concentration, as all participants rated stronger concentrations as more intense than adjacent weaker concentrations (all P’s < 0.001). These data indicate that both groups were able to accurately discern the intensity of the four different concentrations of amyl acetate (Figure 1). No effect of nostril, sex, group-by-sex interaction, or group-by-concentration interaction was observed (Figure 2).

### 3.5 Pleasantness Ratings

Analysis of pleasantness ratings revealed a significant main effect of concentration in control subjects (all P’s < 0.02). In patients with schizophrenia, only scores assigned to the weakest concentration of amyl acetate concentration were significantly different from the others (P < 0.05). A statistically significant group-by-concentration interaction was observed (P < 0.001), and no effect of nostril was observed. As seen in Figure 1, controls showed parallel changes in odor pleasantness ratings, with the strongest odor concentration being rated as the least pleasant (P < 0.001). In contrast, patients with schizophrenia rated weaker odors as less pleasant and stronger odors as more pleasant than controls. Statistically, the ratings for the strongest and weakest concentrations were not different (P = 0.27).

**Figure 3:**
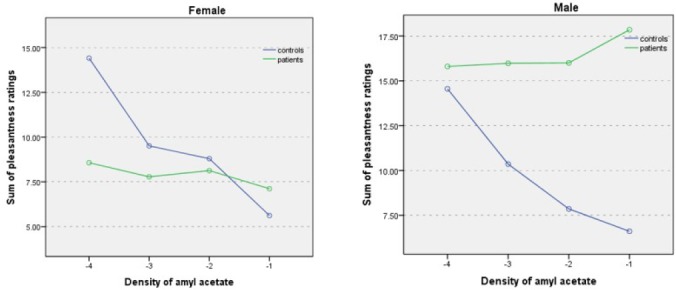
Odor pleasantness ratings by gender for various concentrations (-4.00 to -1.00 log vol/vol) of amyl acetate in schizophrenic patients and controls

In patients with schizophrenia, female subjects’ ratings of pleasantness followed the same trend as control subjects, whereas male patients’ ratings showed an opposite trend: male subjects tended to rate even very strong concentrations as pleasant (Figure 3). The group-by-sex-by-concentration interaction was statistically significant (P = 0.01). Post-hoc MANCOVAs of pleasantness ratings between men and women with schizophrenia in which BPRS, and positive and negative scales of PANSS were used as covariates did not alter the observed sex differences in ratings of odor pleasantness.

### 3.6 Illness Characteristics and Olfactory Functions

There was a predominance of positive symptoms on the PANSS scale in fifteen patients with schizophrenia, and a predominance of negative symptoms in twelve patients. Pleasantness ratings were not significantly associated with age, age of disease onset, education level, illness duration, positive or negative rating on the PANSS, or BPRS score.

When patients were subdivided according to medication status (i.e., SGAs versus FGAs and SGAs), there were no significant main effects or interactions of medication status found. We observed a significant association between chlorpromazine equivalent dosage and pleasantness ratings of the strongest odor concentration (r = −0.40, P = 0.03), but this association was not observed for the other odor concentrations. We defined Δ pleasantness rating and Δ intensity rating as the difference in ratings that participants gave to the strongest and weakest odors. ANOVA of Δ pleasantness rating and Δ intensity rating in male and female patients showed a significant difference between them (P = 0.0001, P = 0.004). Post-hoc analysis showed that only male patients with schizophrenia rated pleasantness scores differently from male and female healthy subjects (P = 0.0001). However, post-hoc analysis of Δ intensity rating revealed that male and female patients were significantly different from male and female healthy subjects, respectively (P = 0.04, P = 0.05). The Δ pleasantness rating was associated with a difference between positive and negative scores on the PANSS (r = 0.36, P = 0.04). Conversely, the Δ intensity rating was associated with a positive score on the PANSS (r = −0.50, P = 0.004), meaning that patients with positive symptoms rated the weakest odors significantly more intensely than other subjects. The illness characteristics of male and female patients are summarized in Table 2.

## 4. Discussion

To our knowledge, no previous study has assessed olfactory function at both threshold and supra-threshold levels. Here, we assessed olfactory function at both levels in patients with schizophrenia through a series of unirhinally administered tests. In our experiments, we did not observe any difference in olfactory processing when testing both of a subject’s nostrils, which suggests that dysfunction in olfactory processing could be occurring in both brain hemispheres. We did not observe a significant effect of drug treatment on olfactory processing, even though a previous meta-analysis (Moberg et al., 2014) indicated that patients that were on a regimen of first generation antipsychotics had significantly greater olfactory dysfunction than those on a regimen of second generation antipsychotics.

Consistent with previous studies (Moberg et al. 1997; Ugur, Weisbrod, Franzek, Pfuller & Sauer, 2005), our results indicate that longer duration of illness is associated with impaired olfactory threshold. We also found that patients with increased positive symptoms have better odor detection sensitivity. This finding is similar to those in a study by Moberg et al. (2006), and is hypothesized to reflect increased vigilance toward external and internal stimuli, which is often seen in patients with predominant Schneiderian symptomatology. Though some authors (Kopala, et al., 1993; Sirota et al., 1999) have attributed impaired olfactory threshold detection in patients with schizophrenia to long-term effects of neuroleptic treatment, we did not observe an impact of antipsychotic treatments on olfactory threshold.

Although we used the Ir-SIT to assess smell identification, our results support the conclusions of previous studies (Kopala, Good, Torrey & Honer, 1998; Kopala et al., 2001; Moberg et al., 1997) that used the UPSIT. In the patients with schizophrenia, we have demonstrated an impact of duration of illness on the smell identification test. Smell identification depends on the integrity of the orbitofrontal cortex and the dorsal medial nucleus of the thalamus (Ugur et al., 2005).

In this study, odorant intensity represents potential sources of variation in odor pleasantness. As suggested by Moskowitz, Dravnieks, and Klarman (1976), the relationship between perceived intensity and pleasantness is often more complex, depending on the odorant in question. The correlation between pleasantness and intensity could be positive (e.g. benzaldehyde), negative (e.g. hexaldehyde), complex (e.g. 3-hexanol), or it may exist no correlation at all (e.g. vanillin). We found that healthy individuals rated amyl acetate as more pleasant at weaker concentrations and increasingly unpleasant as the concentration increased, similar to what has been observed previously (Henion, 1971; Moberg et al., 2003). Our findings surrounding ratings of pleasantness and intensity are in agreement with previous studies (Crespo-Facorro et al., 2001; Hudry et al., 2002; Kamath et al., 2013; Moberg et al., 2003).

In this study, male patients rated amyl acetate odor as significantly more pleasant at higher concentrations. Although this finding is agreement with the results of Moberg’s study (2003), it could be related to how patients and controls use self-report scales or the fact that the proportion of women was lower in this study. It could be related to, Khan et al. (2007) showed that olfactory pleasantness is both variable across individuals and also flexible within individuals over time. However, the pattern of pleasantness ratings by female patients was similar to control subjects. It could be associated to the fact that the proportion of women was lower in this study. Discrepancies in pleasantness ratings between sexes are consistent with reports that male patients experience more enduring negative symptoms, earlier illness onset, and decreased social, cognitive, and premorbid functioning compared with their female counterparts (Leung & Chue, 2000). In a study of temporolimbic and neocortical gray and white matter volumes in patients with schizophrenia, Gur et al. (2000) found significant sex differences in amygdala volumes. Specifically, while 8% of male patients showed a decrease in volume, 10.5% of female patients showed an increased volume in these structures. It may be the initial processing of the olfactory stimulus at the level of the amygdala that is disrupted in male patients, preventing or degrading further processing in orbitofrontal regions.

In contrast to this study, Kamath et al. (2013) and Hudry et al. (2002) showed that both male and female patients had abnormalities in perceived olfactory pleasantness. One explanation for the discrepancy in findings between our study and the Hudry study is the methods used to assess pleasantness. In the Hudry et al. study, pleasantness scores were calculated as an average of the ratings given to a number of different odor types.

Similar to a study by Strauss (Strauss, Allen, Ross, Duke & Schwartz, 2010), we found that patients with positive symptoms selectively judge unpleasant odors as less unpleasant than control subjects do. Although we did not observe an association between negative symptoms and impaired odor identification, this association has been noted many times (Brewer et al., 2001; Kamath et al., 2013; Malaspina et al., 2002). Additionally, Crespo-Facorro et al. (2001) have suggested that there is an association between psychotic symptoms and increased negative ratings of an unpleasant odor. It is possible that the moderately small sample size we had in this study resulted in lack of detection of a correlation between negative symptoms and judgment of odor pleasantness, and further investigation of this is warranted.

A few limitations of our study should be noted. First, a single odor was used to test intensity and pleasantness ratings. This does not allow us to generalize our findings to other classes of odorants. Second, the patients with schizophrenia we tested were predominantly male, young adults with mild to moderate symptoms and without any co-morbidity. The applicability of our findings to older adults and individuals with a broader range of symptoms merits further study. Third, we could not assess the effect of medication status or type of antipsychotic agent used on olfactory functions. Therefore, future studies in larger groups of patients are warranted.

## 5. Conclusion

In conclusion, we investigated various aspects of olfactory function in patients with schizophrenia and healthy controls. Our results suggest that different smell tests highlight different characteristics of olfactory disruption in patients with schizophrenia. Importantly, our main findings are that (1) patients with schizophrenia have impaired smell identification, (2) patients with schizophrenia have impaired olfactory threshold, (3) male patients with schizophrenia have increased olfactory pleasantness ratings on the amyl acetate test when compared with female patients and controls, (4) patients with schizophrenia did not differ from healthy subjects when rating the intensity of amyl acetate odor, and (5) patients displayed no abnormal laterality in olfactory measures

We suggest investigating other aspects of olfactory function in patients with schizophrenia, and combining future studies with neuroimaging techniques may provide valuable diagnostic and physiological information surrounding this heterogeneous disorder.
